# Forest carbon stock development following extreme drought-induced dieback of coniferous stands in Central Europe: a CBM-CFS3 model application

**DOI:** 10.1186/s13021-023-00246-w

**Published:** 2024-01-03

**Authors:** Emil Cienciala, Jan Melichar

**Affiliations:** 1 IFER – Institute of Forest Ecosystem Research, Cs. Armady 655, 254 01 Jilove U Prahy, Czech Republic; 2 Environment Centre, Charles University, Jose Martiho 407/2, 162 00 Prague, Czech Republic

**Keywords:** Forestry, Adaptation, Bark-beetle, Carbon stock change, Ecology, Mitigation, LULUCF, Model scenarios, Forest management

## Abstract

**Background:**

We analyze the forest carbon stock development following the recent historically unprecedented dieback of coniferous stands in the Czech Republic. The drought-induced bark-beetle infestation resulted in record-high sanitary logging and total harvest more than doubled from the previous period. It turned Czech forestry from a long-term carbon sink offsetting about 6% of the country's greenhouse gas emissions since 1990 to a significant source of CO_2_ emissions in recent years (2018–2021). In 2020, the forestry sector contributed nearly 10% to the country's overall GHG emissions. Using the nationally calibrated Carbon Budget Model of the Canadian Forest Sector (CBM-CFS3) at a regional (NUTS3) spatial resolution, we analyzed four scenarios of forest carbon stock development until 2070. Two critical points arise: the short-term prognosis for reducing current emissions from forestry and the implementation of adaptive forest management focused on tree species change and sustained carbon accumulation.

**Results:**

This study used four different spruce forest dieback scenarios to assess the impact of adaptive forest management on the forest carbon stock change and CO_2_ emissions, tree species composition, harvest possibilities, and forest structure in response to the recent unprecedented calamitous dieback in the Czech Republic. The model analysis indicates that Czech forestry may stabilize by 2025 Subsequently, it may become a sustained sink of about 3 Mt CO_2_ eq./year (excluding the contribution of harvested wood products), while enhancing forest resilience by the gradual implementation of adaptation measures. The speed of adaptation is linked to harvest intensity and severity of the current calamity. Under the pessimistic Black scenario, the proportion of spruce stands declines from the current 43–20% by 2070, in favor of more suited tree species such as fir and broadleaves. These species would also constitute over 50% of the harvest potential, increasingly contributing to harvest levels like those generated by Czech forestry prior to the current calamity. The standing stock would only be recovered in 50 years under the optimistic Green scenario.

**Conclusion:**

The results show progress of adaptive management by implementing tree species change and quantify the expected harvest and mitigation potential in Czech forestry until 2070.

## Background

Forests are increasingly treasured for their wide range of benefits to society. Apart from being a source of wood, they are valued for their other services or functions, including recreation, water retention, providing habitat for biodiversity, soil conservation, and more. Forestry has gradually become an integral part of climate policies within the emission sector LULUCF (Land Use, Land-Use Change and Forestry) due to its potential for mitigating CO_2_ emissions and storing carbon in managed forest ecosystems. The European Green Deal, a recent climate strategy approved by the EU in 2020, incorporates forestry into its climate targets in several ways. It integrates the new EU forest strategy for 2030 with the EU biodiversity strategy, thereby making the objectives of biodiversity conservation and climate action more coherent.

Despite mounting evidence of declining carbon sink in European forestry in recent years [1, 2] and increasing disturbances affecting forests in Europe [3], the climate ambition for forestry in the EU has progressively increased during the negotiation process: the current expectation for the Land-use, Land-use Change and Forestry (LULUCF) sector is to achieve a greenhouse gas (GHG) emission offset of 310 Mt CO_2_ eq.by 2030 (Regulation EU 2023/839, [4]). Although not yet adopted, there are intensive negotiations for the EU commitment that the LULUCF sector should at least compensate for greenhouse gas emissions from the Agriculture sector (mainly non-CO_2_ emissions from livestock and manure management) within the joint sector called Land (Agriculture and LULUCF) by 2035. This would follow the earlier methodological concept of AFOLU (Agriculture, Forestry and Other Land Use; [5]).

Evidently, the primary focus for delivering the mitigation effects lies on the existing managed forests in the EU and its individual Member States [4, 6]. Within this context, the Czech Republic appears to be particularly vulnerable. In recent years (2018–2021), the Czech forestry sector has been one of the few in the EU that generated CO_2_ emissions. This is mainly due to the decline of predominantly Norway spruce-dominated stands caused by an unprecedented drought [7, 8] that induced the extreme bark beetle calamity since 2018 (initiated earlier—in 2014/2015—in Eastern part of the country). As a result, the required sanitary harvest volume extremely increased from about 4.5 Mm^3^ in 2014 to almost 34 Mm^3^ in 2020 [9], which significantly exceeded the current growth rate. The total merchantable wood volume harvest (under bark) more than doubled relative to earlier levels, reaching the maximum of 35.8 Mm^3^ in 2020, with sanitary harvest comprising 95% of that total volume. This development transformed the Czech forestry sector, which previously offset about 6% of the country's annual GHG emissions, into a net source of emissions in the past few years since 2018 [10]. In 2020, when the bark beetle calamity reached its peak, emissions from forestry represented 11% (9%) of the country's overall emissions excluding (including) the positive emission effect of harvested wood products (HWP).

European forests, particularly in Central-European conditions, are subject to intensive management. Hence, forest management is the essential tool to effectively steer the necessary adaptation of the vulnerable forest ecosystems into a more resilient natural systems that would cope better with changing growth conditions and possibly benefit from them. Although the current forest adaptation strategies better integrate the spectrum of forest functions and services in their targets [11, 12], the implementation progress and quantitative impacts remain uncertain. Hence, assessing development of forest resources under alternative forest management scenarios is needed to apprehend the likely effect of adaptative management on wood resources, forest structure and carbon budget.

The estimation of ecosystem carbon stock changes under conditions of significant or extreme annual disturbances caused by both biotic and abiotic factors, which necessitate extensive mandatory forest management sanitary interventions, is a challenging task. It requires the use of appropriate estimation tool capable of assessing changes in all carbon pools with the forest ecosystem (living biomass above and below-ground, dead organic matter including litter and deadwood above and below-ground, and soil) for the desired period, spatial scale, and temporal resolution.

In this contribution, we used the Carbon Budget Model of the Canadian Forest Sector (CBM-CFS3, [13, 14], here also denoted as CBM) to estimate forest carbon stock and its changes for the period 2018–2070 using a range of likely management and disturbance scenarios. In that period, the years 2018–2021 represent the known forest intervention regime, while 2022–2070 is the true projection period under specific forest management and spruce decline scenarios. CBM was initially developed to meet the operational-scale forest carbon accounting needs of forest managers and analysts in Canada [13], but it was soon adopted and calibrated for forestry and carbon accounting purposes in European counties [15–19]. Specifically important was creation of the European database describing the country-specific calibration of the model [20]. For the conditions in the Czech Republic, the model was earlier specifically calibrated and used for forestry-related estimates in the LULUCF sector in the Czech national GHG emission inventory [21, 22]. The decision to implement CBM as a tier-3 estimation methodology for the Czech forestry sector in the national GHG inventory under UNFCCC according to the IPCC guidelines [23, 24] was driven by the fact that only such approaches could adequately capture carbon stock changes in all forest ecosystem carbon pools. These respond dynamically to the intensive management interventions associated with sanitary measures addressing the large-scale decay of coniferous stands in the country [21].

The aim of this study was to quantify the expected effects of adaptive forest management on forest resources in terms of carbon stock change and CO_2_ emissions using a methodology that is coherent with the Czech national GHG emission inventory for the forestry sector [22]. For this, we used a set of four spruce forest dieback scenarios and associated harvest structure, ranging from an optimistic (Green) scenario assuming a rapid improvement of the current calamity situation, to progressively more pessimistic scenarios (Red, Black) assuming a more sustained and/or recurrent (Black rep.) calamity period. Other analyzed effects of the adaptive forest management included changes in tree species composition, development of harvest potential, and changes to forest age structure. This information is desired by the policymakers to assess the progress of implementing adaptation measures including quantification of their effects at regional and national scales.

## Results

### Overall carbon balance and emissions

**Fig. 1:**
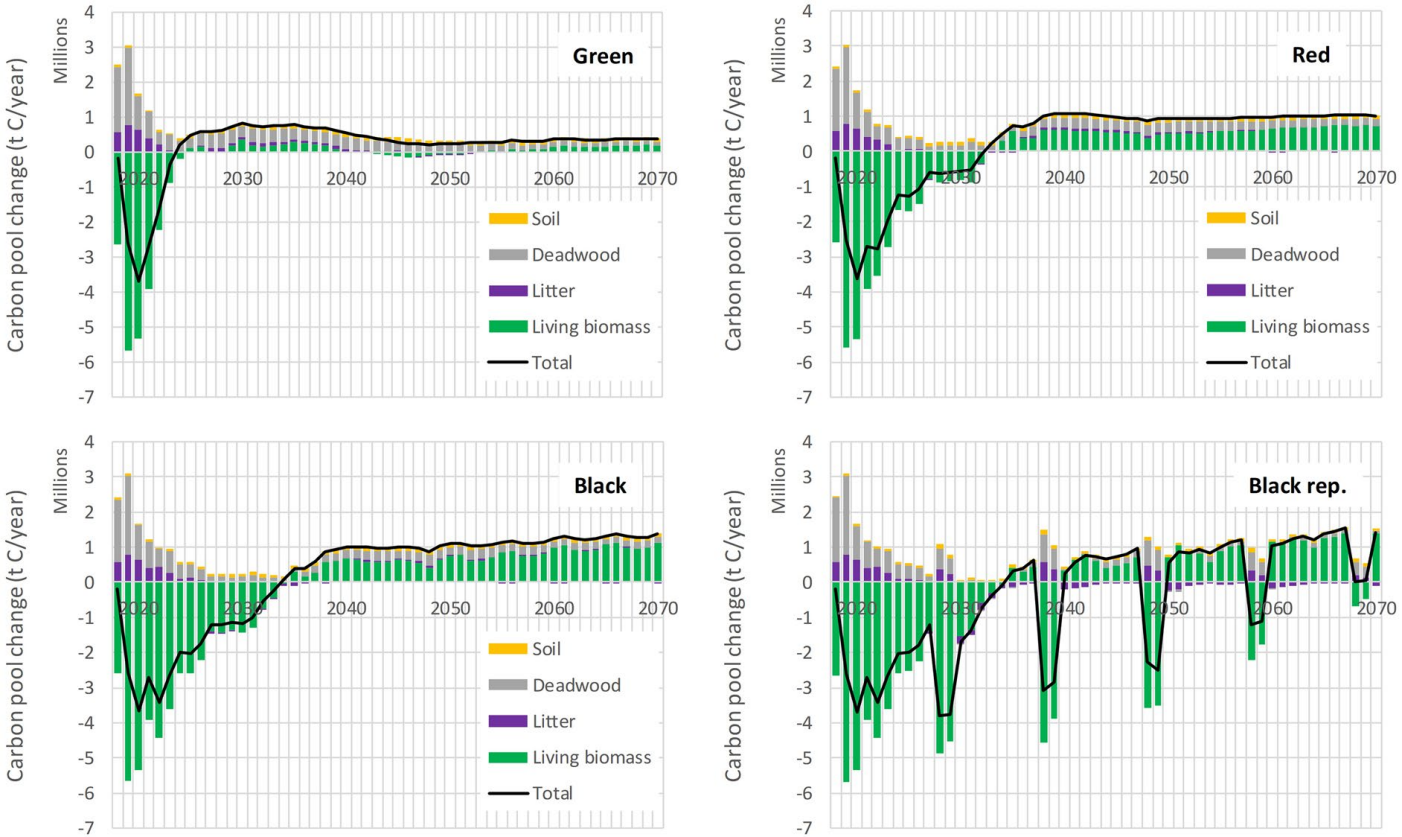
Carbon pool changes for the four scenarios (Green, Red, Black and Black rep.) for the period 2018–2070. Four carbon pools (Living biomass, Litter, Deadwood, Soil) are shown by bars, together with the net total carbon stock change (Total) by solid black line

Figure 1 illustrates the carbon balance of the country, aggregated across all NUTS3 regional units, for all four scenarios, based on individual carbon pools. The trajectories of the projected carbon balance are nearly identical for all scenarios during the period 2018–2021, as the input activity data used for estimation by CBM align with the known (reported) observations by the Czech Statistical Office [9]. However, the carbon balance diverges during the period of 2022–2070, reflecting the scenario-specific forest management interventions and assumptions.

The Green scenario presents an optimistic outlook, showing a rapid recovery from the ongoing bark-beetle calamity in the country. It reaches a carbon balance break-even point in 2024 and continues to accumulate carbon thereafter. The development until 2070 demonstrates the sustainable utilization of available wood resources, with wood removals of approximately 4.2 Mt C/year, corresponding to a merchantable wood volume of about 17 Mm^3^ annually. The carbon stock changes shown in Fig. 1 also include extraction of harvest residues with an intensity of 20–25%.

In contrast, the more pessimistic Red, Black, and Black rep. scenarios depict a longer recovery trajectory for the forest carbon balance of the country. Following the recent extremes observed in 2018–2021, the carbon balance becomes positive just after 2030 under the Red scenario, with the Black and Black rep. scenarios experiencing a slight delay (Fig. 1). For the Black rep. scenario, the carbon balance periodically turns negative, reflecting prescribed disturbance (recurring bark-beetle outbreaks combined with prolonged drought), occurring once per decade during the projection period. However, apart from these periodic events, the positive carbon balance approaches 1 Mt C annually for the period after the stabilization of the current calamity, continuing until 2070. The amount of sequestered carbon under the Red and the Black scenarios reflects a somewhat reduced harvest demand compared to the Green scenario (Table 4) that is implemented after the cessation of the bark-beetle calamity period.

**Fig. 2:**
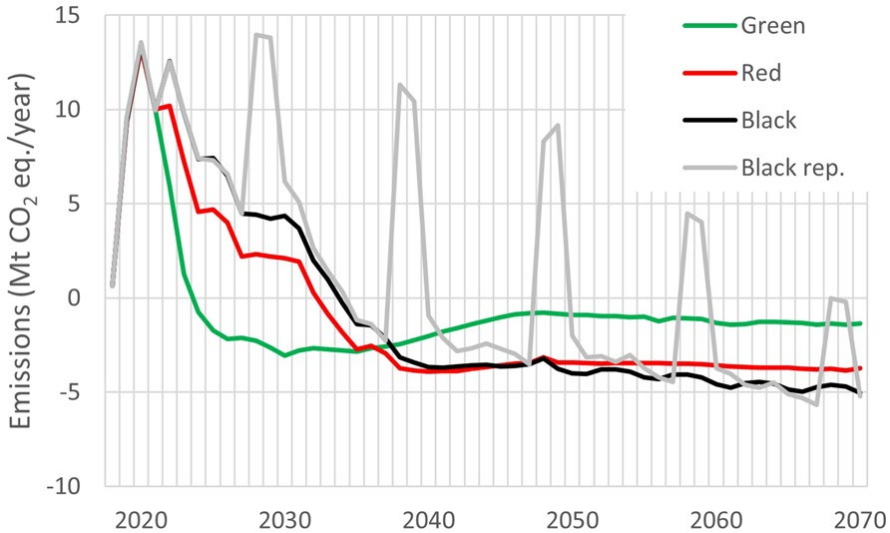
Emissions from forestry under four scenarios (Green, Red, Black, Black rep.) for 2018–2020. Quantities for known (reported) years 2018–2021 are practically identical, projections (2022–2070) are scenario-specific. The negative values represent a net sink of CO_2_ emissions

Figure 2 shows the scenario-specific carbon balance expressed in terms of CO_2_ eq. emission/removal units. For the initial years 2018–2021 that run on the observed/reported input data, the average annual emission contribution for these 4 years across all scenarios was 8.37 Mt CO_2_ eq./year. This is fully coherent with the corresponding mean 9.34 Mt CO_2_ eq./year from the GHG emissions reported by the Czech Republic for the category 4.A Forest land in its latest national GHG inventory report (NIR) under UNFCCC ([10]). The observed minor quantitative differences reflect a better handling of standing dead trees (postponed harvest based on the reported data) and revised extraction intensities of harvest residues for salvage and planned harvest, which has not been correspondingly implemented in the NIR yet [10, 22].

**Fig. 3:**
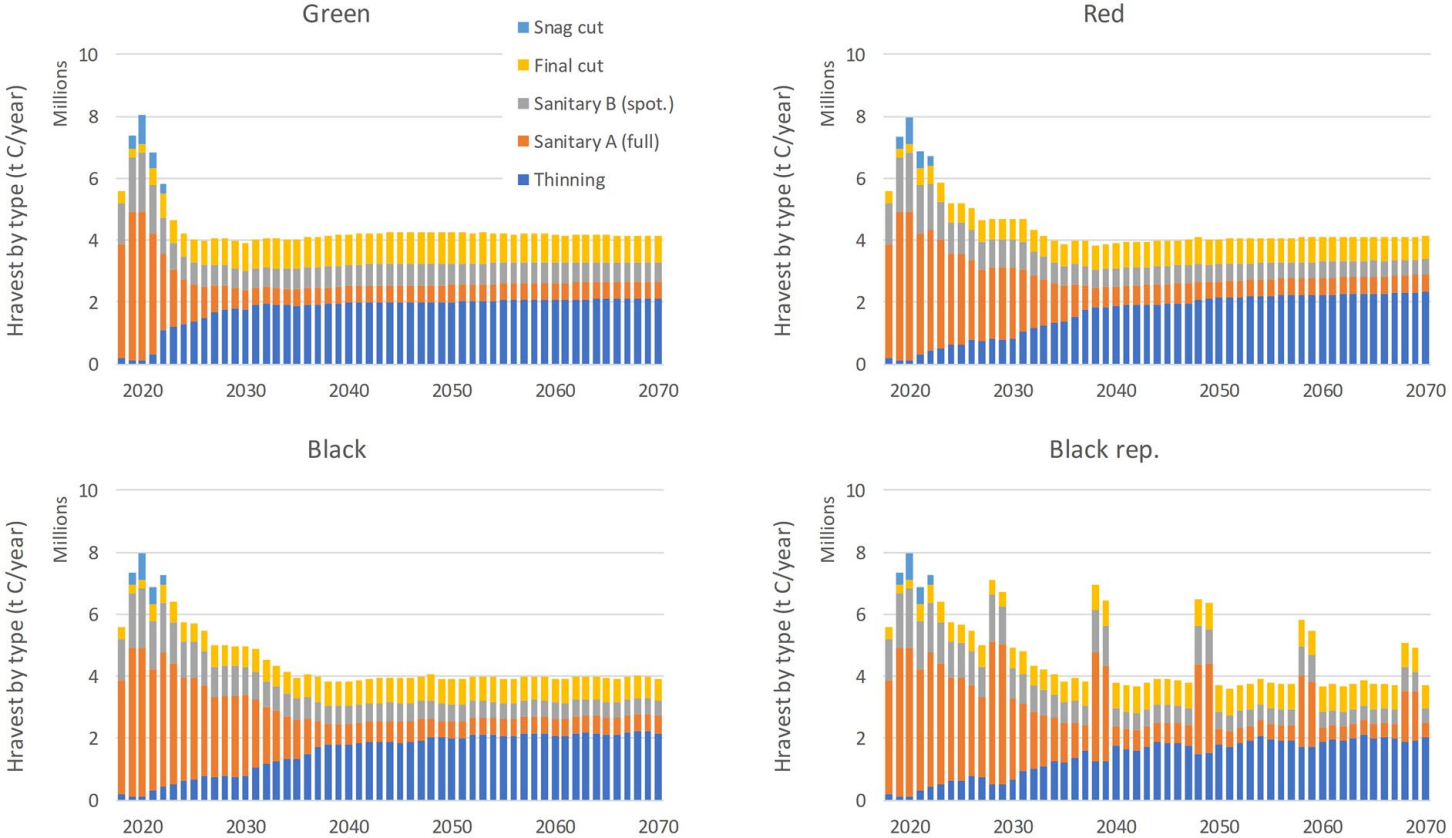
Actual harvest by types for the four scenarios (Green, Red, Black and Black rep. in the respective panels) and the period 2018–2070. Sanitary A (full), B (spot.) represent two types of sanitary loggings (Table 5), Thinning and Final cut are planned harvest interventions, Snag cut (applicable only for 2018 2022 applying one year delay) is felling of snags (dead standing trees). See Table 2 for details on disturbance types related to harvest

The following part of the projection period from 2022 to 2070 reflects the scenario-specific drivers and assumptions. The optimistic Green scenario shows a rapid decline of the emissions and reversing to a sink of emissions prior year 2025 with a sustained carbon accumulation for the rest of the projection period. It would reach over −2.5 Mt CO_2_ eq./year and later stabilize at the level of about 1 Mt CO_2_ eq./year. The emission decline from the lately (2021) observed level is slower for the more pessimistic Red and Black scenarios, which would turn the forestry sector into a sink after the year 2030. Thereafter, the two scenarios show a notable sink of CO_2_ emissions for the rest of the projection period at the level at around −3.5 and −4. Mt CO_2_ eq./year, respectively. This corresponds to a more conservative harvest demand used in these two scenarios, which translates to ca. 16 Mm^3^ of merchantable wood annually (Table 4). Finally, the hypothetical Black rep. scenario including a reoccurring calamity disturbance shows the emission peaks reflecting the available biomass that is extracted dominantly by salve logging interventions (Dist. 3a, 3b; Table 2). The peaks gradually decline as the carbon pool in living biomass of spruce stands gradually diminishes as the excessive periodical harvest demand set for spruce apparently cannot be met (Fig. 3).

### Allocated harvest, its structure, and effects on growing stock

Figure 3 summarizes the scenario-specific harvest by individual disturbance types representing wood removals. These are thinning and final cut as the planned forestry interventions under classical forest management in Central Europe. The sanitary logging resulting with clearcut (Dist. 3a; Table 2) or without clearcut (Dist. 3b; Table 2) are the mandatory management interventions that must be prioritized over the planned management interventions in compliance with the Czech Forest Act to minimize spreading of forest disturbance and minimize environmental and economic impacts. The maximum harvest for all scenarios reached 8 Mt C/year in 2020. This corresponds to the base harvest of almost 35.8 Mm^3^ of extracted merchantable wood volume as reported for the country [24]. The harvest levels are practically identical for the four scenarios used here during the initial four years of the simulations (2018–2021) as for magnitude and harvest structure. Thereafter, the development is scenario-specific.

For the Green scenario, harvest levels decline quickly and reach a level of about 4 Mt C/year in the second half of this decade and stabilize at just under 4.2 Mt C/year for the rest of the projection period until 2070. For the Red and Black scenarios, reduction of harvest levels proceeds notably more slowly, and harvest stabilizes only in 2030s (Fig. 3). It levels at around 4 Mt C/year towards the end of the simulated period. For the Black rep. scenario, where the requested repeated sanitary harvest levels are defined by the average extracted volumes by species groups as of 2018/2019, the allocated harvest is progressively smaller due to the limits in biomass available to harvest for the spruce species category in several NUTS3 regions. The harvest demand is increasingly not met for this scenario, with the overall deficit (generally attributed to spruce) over 20% in the last two decades and increasing to over 45% in the last recurring calamity episode. In contrast, harvest demand is generally met for all other scenarios, except the Black scenario developing a slight deficit in allocated spruce harvest of about 5% towards the end of the projected period. This trend also explains the increasing CO_2_ sink for the Black scenario relative to the Red scenario observed in Fig. 2.

All scenarios show a specific pattern of harvest structure (Fig. 3), showing a gradually rising contribution of planned harvest interventions, i.e., specifically thinning and final felling. In accordance with that, the share of sanitary logging declines to a more acceptable share of about 25–29% of the total harvest for the last decades under the Green, Red and Black scenarios. This corresponds to the share of sanitary logging observed prior to the current calamity in early 2010s.

Not shown in the graphs is the contribution of individual species to the allocated harvest. In the most extreme observed years 2019–2020, the share of broadleaved tree species was only 6% of the total harvest. This proportion increased to about 40% and 46% for the last simulated decade during 2060s, the last decade of the projected period for the Green and the Red scenarios, respectively.

**Fig. 4:**
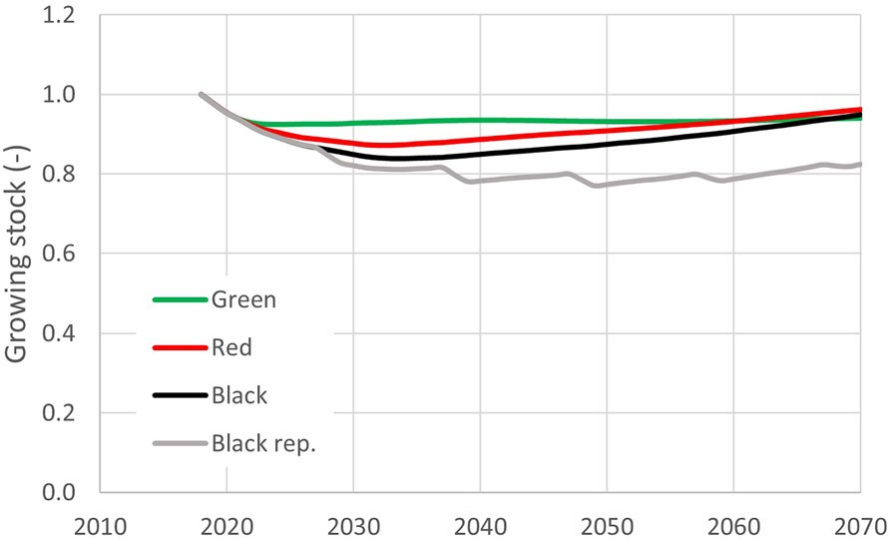
Scenario-specific effect on growing stock (living biomass) during the projection period on relative scale with respect to initial conditions (year 2018)

The effect of the all-time high harvest levels during the calamity years is detectable on the mean growing stock (Fig. 4). After the initial decline following the currently observed calamity and extremely elevated harvest (2018–2021), the stabilization pattern reflects the imposed harvest intensity thereafter. The Green scenario shows a stabilization of the living biomass carbon pool already since the mid-period of the current decade. The decline of living biomass carbon pool is more pronounced for the Red and the Black scenarios that apply higher sanitary harvest levels with a following recovery for the rest of the simulation period. Finally, the pessimistic Black rep. scenario with reoccurring pattern of tree dieback requiring sanitary logging would result in a more severe decline of living biomass carbon stock leveling at about 20% of the initial biomass carbon observed for 2018. Compared to that, the living biomass carbon pool under the other three scenarios would recover to about 95% in 2070 as compared to the original value observed in 2018.

### Changing species composition and stand age structure

**Fig. 5:**
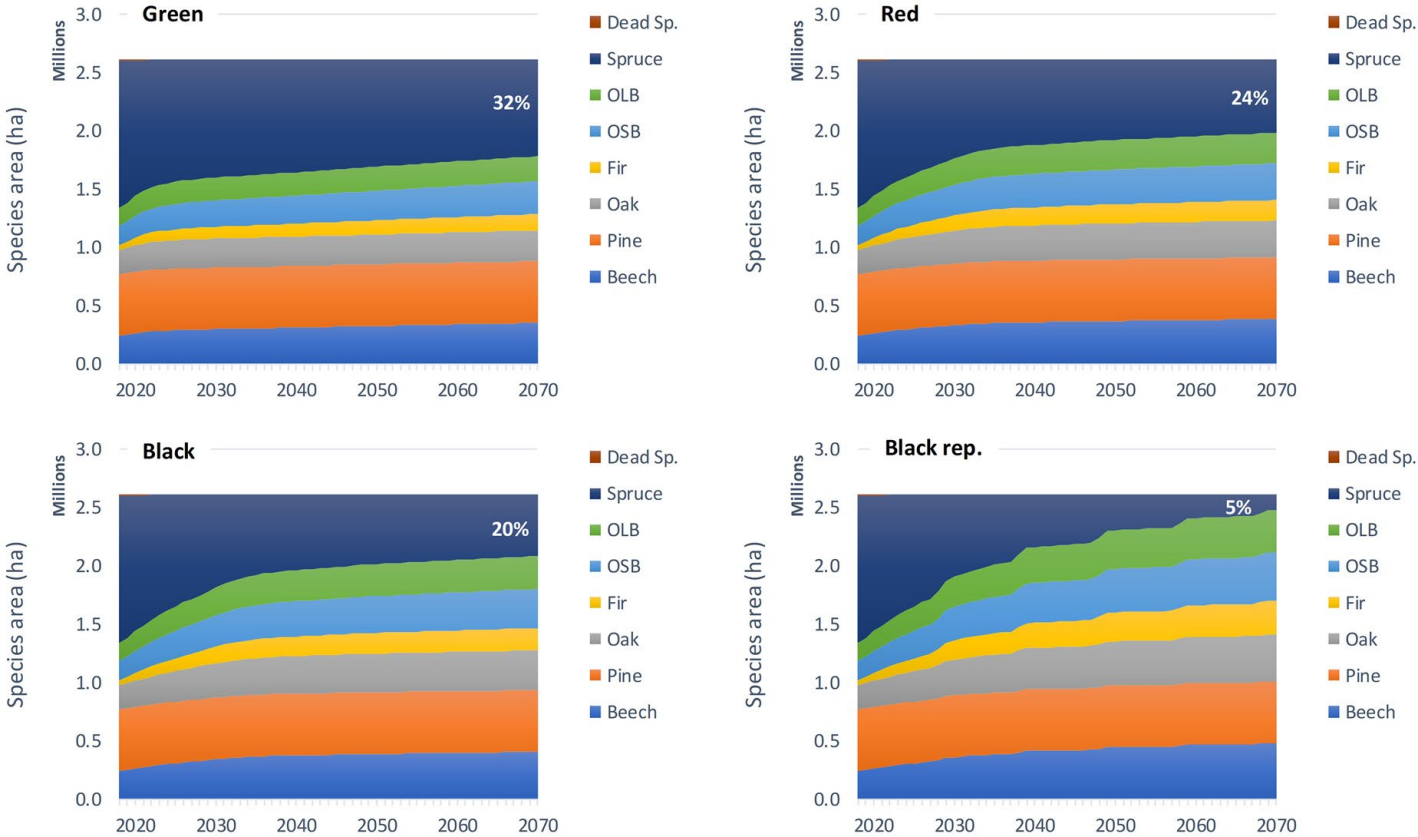
Species change for the four scenarios (Green, Red, Black and Black rep.) and the period 2018–2070. The percentage shows the remaining share of vulnerable Norway spruce at the end of the period in 2070. Species groups—OLB (other long-lived broadleaves), OSB (other short-lived broadleaves), see Table 5 for details on species grouping

The key indicator of the adaptive forest management scenarios is the change in species composition. It is summarized in Fig. 5. The most critical is the reduction and replacement of unstable spruce-dominant stands. The share of these would decline from the initial representation of 50% in 2018 (Table 5) to 32% in 2070 under the Green scenario. The share of spruce would decline more notably under the Red and Black scenarios, reaching 24 and 20% share in forest area. Finally, the most pessimistic Black rep. scenario imposing the most severe reoccurring sanitary harvest would limit the spruce representation to only 5% in 2070. This decline is compensated for by an increase of other desired tree species—mainly the valuable broadleaved tree species and fir species group (Fig. 5).

**Fig. 6:**
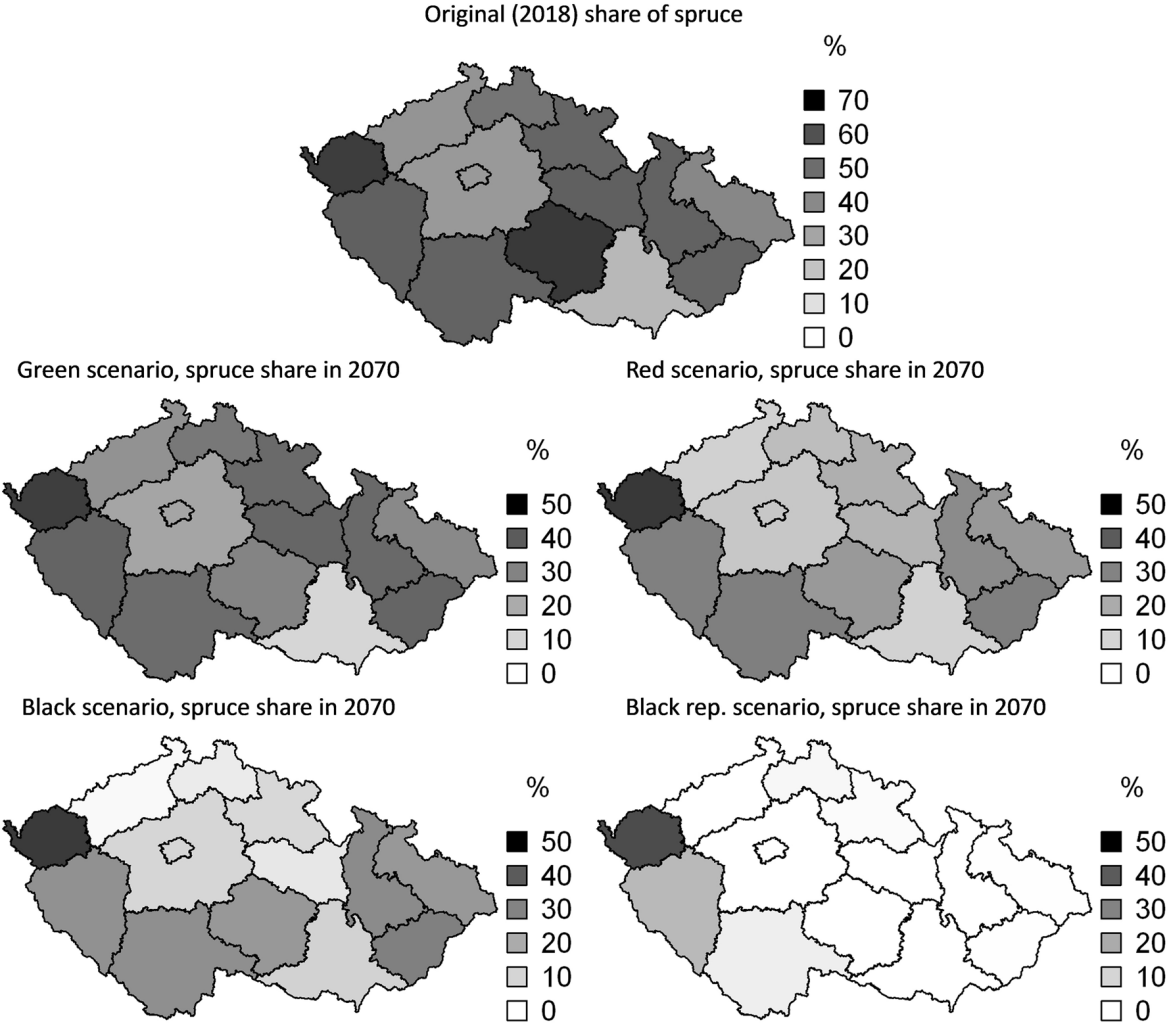
Change in spruce share for the four scenarios during period 2018–2070. The percentage shows the remaining share of vulnerable Norway spruce at the end of the period in 2070 relative to the original state as of 2018 (top figure)

The decline of spruce share is region-specific and can be spatially visualized using cartograms (Fig. 6). Besides the Prague region with a negligible spruce growing stock, the decline in the vulnerable spruce share between 2018 and 2070 ranges from 10% (e.g., Ústecký region for the Green scenario) to almost 100% (e.g., Vysočina region for the Black rep. scenario) across the individual NUTS3 regions and scenarios (Fig. 6). Evidently, the change in species composition is related to the initial conditions (spruce share at the beginning of simulation in 2018) and the scenario-specific harvest intensity.

**Fig. 7:**
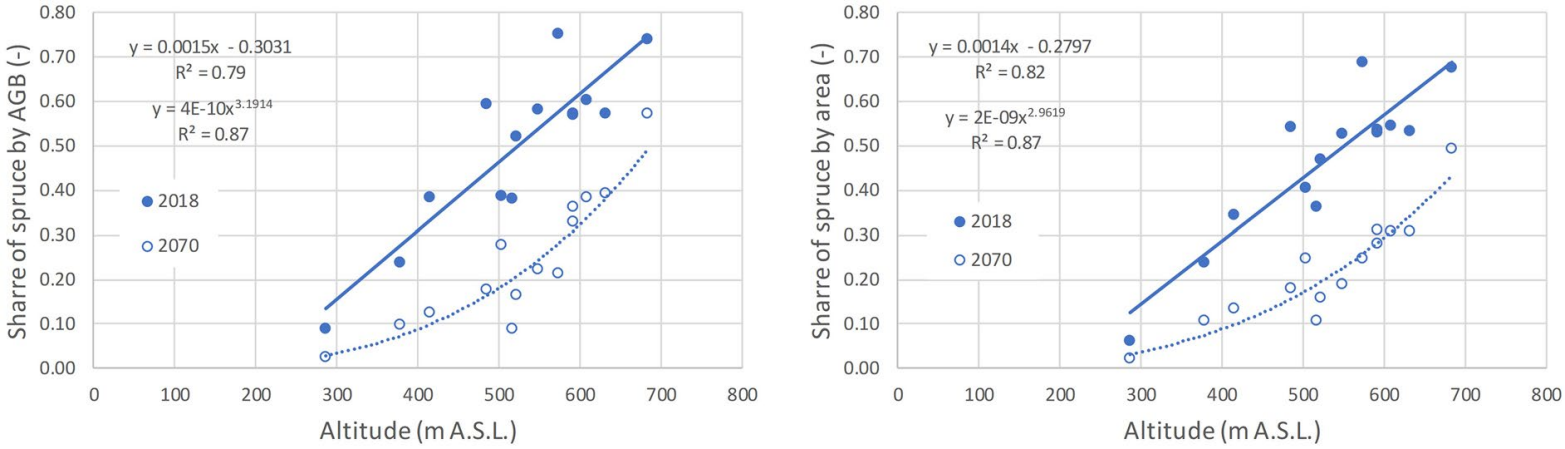
Shift in spruce share for the Red scenario expressed either in the aboveground biomass share (AGB, left) or the species area share (right) by individual NUTS3 regions. The condition in the initial year 2018 (filled symbols) and that at the end of simulated period in year 2070 (open symbols) is shown. The relation of spruce share to the mean altitude of forest areas in NUTS3 regions is shown, approximated by linear (2018) or power function (2070)

Next, the change reflects the habitat suitability for spruce that is determined by altitude—the most severe decline is observed in the regions with the lowest mean altitude, which reflects the temperature and moisture gradient in the landscape. This relationship is explicitly explored in Fig. 7. The higher loss of spruce is observed for the regions with the spruce stands of the low and middle locations at around 500 m A.S.L. The general relationship becomes non-linear, reflecting a progressively tighter climate-dependency of habitat suitability for spruce during the simulated period—expressed either by area or growing stock volume share of spruce (Fig. 7). The average altitude of forest areas for the individual NUTS3 regions is listed in Table 3.

**Fig. 8:**
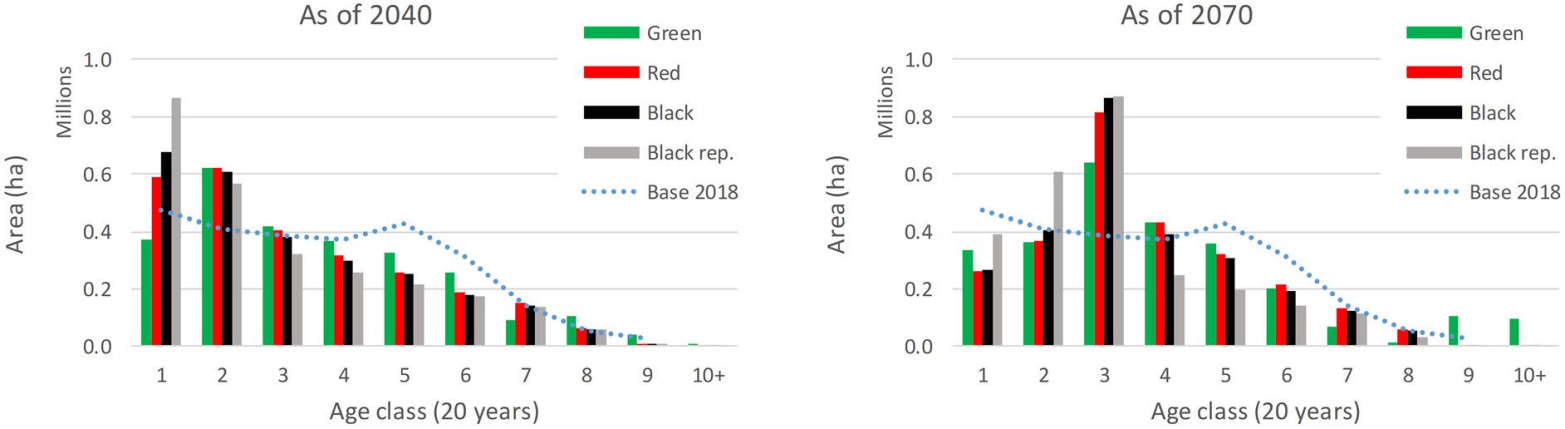
Development of forest age structure under the four scenarios shown for the years 2040 (left) and 2070 (right) relative to the initial distribution as of 2018

The intensive harvest responding to the current calamity outbreak in the country affects the age structure of the forest growing stock. The projections under the four scenarios carry over the legacy of the current extreme disturbance period that is pronounced in the following decades (Fig. 8). For all scenarios, there is a significant increase, relative to the initial state in 2018, in stand area of the 2nd and 3rd age class around 2040 and 2070, respectively. There is also a decrease in representation of mature forest stands of the 5th and 6th age class (80–120 years) noticeable in both 2040 and 2070 as a legacy of the increased sanitary felling performed earlier. This is especially noticeable in the Black rep. scenario. Specifically for the Green scenario towards the end of the simulation period, there is a visible increase in the representation of old-aged stands (above 160 years). This is due to the implemented measure to preserve the healthy old stands above 120 years (conifers) and 140 years (broadleaves) for biodiversity and excluding them from the planned harvest. Such old-aged stands are missing in the case of pessimistic scenarios at the end of the simulation period.

**Fig. 9:**
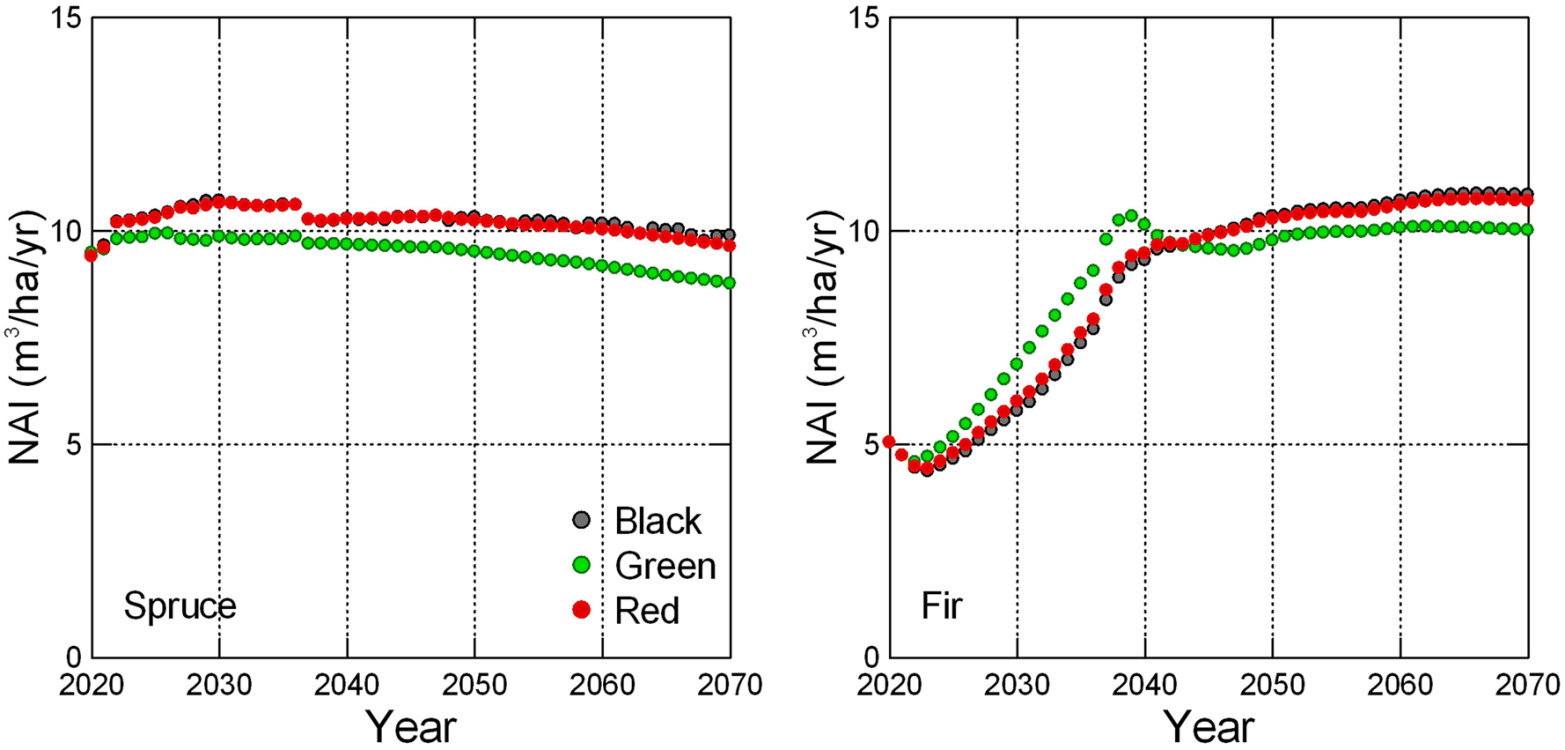
Net annual increment (NAI) for Spruce (left) and Fir (right) species groups and scenarios, (except Black rep.) for the period 2020–2070

Due to the imposed change in species composition, the development of age structure varies depending on the species. The most notable change observed is the declining share of spruce during the simulated period. Conversely, other species groups would see an increase in their representation. This shift in species composition would impact the net annual increment (NAI), as demonstrated by the example of spruce and fir tree species groups (Fig. 9). NAI for the productive spruce tree species shows a slight declining trend from the level close to 11 m^3^/ha to about 10 m^3^/ha or less across all scenarios. This trend reflects the aging of the standing growing stock and the increased proportion of less-productive older stands. This is mostly manifested for the Green scenario that also includes retention of older tree stands (Table 4). On the other hand, fir tree species group benefits from enhanced afforestation efforts, and its NAI would soon reach the mean NAI level like that of spruce, eventually surpassing it towards the end of the simulated period due to a more favorable development of the age structure.

## Discussion

### Projected emissions and the commitments under the EU LULUCF regulation

The current calamity experienced in the country since mid-2010s has no recorded historical analogue to its scope and devastating effect on the Czech spruce-dominated forests [8]. This is unfortunate also in the context of the adopted commitments under the EU LULUCF regulation [4, 6]. The estimated carbon balance of the four scenarios (Figs. 1, 2) indicates the overall uncertainty with respect to the cessation of the current historically largest calamity experienced in Czech Forestry, which also makes the near-term development of the resulting emissions uncertain (Fig. 2). Specifically important will be the period towards the end of the current decade. This is because of the accounting setting adopted by the EU LULUCF regulation 841/2018 [6] for 2021-2025 and its newly adopted revision 839/2023 [4] that includes the emission reduction goal of −310 Mt to be achieved by the Union’s LULUCF sector in 2030 with the specified targets for the individual Member states. Since forestry largely dominates within the LULUCF sector in terms of the annual carbon stock changes and associated fluxes of CO_2_, most of the expected emission offset is expected just by forestry. The projection indicates that the adopted accounting scheme and expected emission reduction commitment will be very challenging for the forestry sector in the Czech Republic.

It should be noted that the emission accounting for the pre- and post-2025 period largely differs. For the period 2021–2025, the emissions and removals from forestry are in individual EU Member States accounted towards the so-called Forest reference level (FRL), which is based on country-specific projections based on the forest performance during 2000–2009 period. This setting proved to be technically overly complex and hence untransparent [17], providing apparently unjustified benefits to some countries and penalizing others (see, e.g., Fig. 12 in [25]). The Czech Republic belongs to the latter group. Despite the evidence of the alarmingly progressing forest calamity expected to affect country’s compliance for the post -2020 period, the EU Commission adjusted the proposed FRL by the Czech Republic in its delegated act [26], disregarding the arguments by the Czech Republic on a necessity to consider the observed harvest trend in the reference period 2000–2009. This set the Czech FRL at a clearly unrealistic level of −6.137 (−4.739) Mt CO_2_ eq./year including (excluding) the contribution of Harvested wood products (HWP). Hence, this national GHG accounting benchmark became even more stringent than the earlier Forest management reference level (FMRL) of −4.686 Mt CO_2_ eq./year, which was used under the Kyoto protocol forest management accounting for the second compliance period 2013–2020. The consequence of this unfortunate setting is a massive deficit of CO_2_ removal units expected to be accounted for in the period 2021–2025 using the imposed FRL. Using the results of the optimistic Green scenario, the estimated CO_2_ balance for the forests in the country (Fig. 2) may cumulatively reach 14.6 Mt CO_2_ eq. for the period 2021–2025. The country-specific FRL adds additional 23.7 Mt CO_2_ (excluding HWP contribution). Hence, the expected total deficit to be accounted for the Czech Republic would fall close to 38 Mt CO_2_ eq. for the period 2021–2025. These estimates exclude the potential contribution of HWP, other land use categories and land use conversions to forest land that should be included in the LULUCF commitment. This would slightly improve the total estimates, as the average annual emission reduction from HWP and afforestation reached jointly −1.75 Mt CO_2_ eq., making a potential total contribution of −8.7 Mt CO_2_. This would still result in an accumulated deficit of about 29 Mt CO_2_ under the optimistic and probably most realistic Green scenario for this accounting period. There is a marginal potential to ease the effect of the current FRL using a technical correction during accounting. This would be justified due to the recently enhanced national GHG emission reporting covering explicitly all carbon pools using the nationally calibrated CBM model [21]. The revised LULUCF regulation [4] includes two articles designed to ease the effects natural disturbances in emission accounting for 2021–2025. However, both remain unusable for Czech Republic: Art. 10 (disturbance provision) explicitly prohibits exclusion of salvage logging from accounting, while the use of Art. 13(4) is dependent on a surplus of CO_2_ credits generated by LULUCF in EU, which is unlikely to happen [27].

For the period 2026–2030, the FRL accounting setting is not applicable anymore for the European Member States. It is replaced by a more robust and fair target setting adopted in the new EU LULUCF revision 2023/839 [4], based on the sector performance assessed from the emissions and removals in 2021–2023 as reported in the 2025 NIR submissions. For the pentad 2026–2030, the GHG emissions under the Green and the Red scenarios quantified in this study (Fig. 2) show the total balance for Czech forestry as −12.3 and 12.8 Mt CO_2_ eq., respectively. This translates to −2.5 and 2.6 Mt CO_2_ eq. annually for the two scenarios, which is the range that includes the expected target of −1.23 Mt CO_2_ eq. set in the revised LULUCF regulation for Czech forestry for year 2030. Hence, this emission target may already be achievable by the country, taking also into account the likely additional offsets by afforestation and HWP.

Important to note is the contribution of other carbon pools besides living biomass in the total carbon budget, which is shown important both in short- and long-term perspective (Fig. 1). It is apparent that the importance of deadwood and litter increases in the periods of elevated harvest, when it partly offsets the losses by extracted aboveground living biomass held in merchantable wood. Besides the fraction of harvest residues, the accumulation of deadwood includes below-ground component of harvested trees and unprocessed standing deadwood in case of technical harvest limitations (as was the case for years 2018–2021 in the country) to address the excessive sanitary harvest demand. Next, intentional accumulation of deadwood and dead organic matter in general is commonly proposed to enhance biodiversity, prevent nutrient degradation and to increase carbon storage [28]. All these objectives are well substantiated in the context of Czech forestry, where soil remains notably disturbed [29, 30] and the average amount of deadwood remains low [31]. Hence, increasing the carbon pool of dead organic matter and indirectly soil represents—at least for medium term [32]—additional mitigation potential, besides enhancing biodiversity, for forests that have been intensively managed earlier.

### Long term outlook for the Czech forestry sector and adaptation issues

Long-term outlook is important in assessing the implementation of adaptive forest management, quantifying the key characteristic of changing forest resources under changing growth environment. Our projections beyond the 2030 horizon until 2070 show the possible stabilization pathways under the tested scenarios both in terms of the mitigation function and increased stability and resilience of forest resources.

The optimistic Green scenario preserves the largest share of spruce for the simulation period and permits the above-average harvest relative to the conditions prior to the current bark-beetle calamity in the country. Specifically, the mean annual base harvest reached 15.8 Mm^3^ of merchantable volume (under bark) and 16.6 Mm^3^ considering additional extraction of harvest residues, as assessed for the period 2001–2015 [9, 22]. Hence, the target annual harvest of 16 or 17 Mm^3^ attainable under the Green, Red and Black scenarios (Table 4) should meet or exceed these levels, while preserving sustainability and successively increasing growing stock and carbon accumulation, except the hypothetical Black rep. scenario (Fig. 4).

This is a positive outlook for forestry because most of the vulnerable spruce stands would be replaced by more resilient fir tree species and broadleaves. The fir species group can partly offset the missing spruce wood production, which is largely favored by the wood-processing industry [33]. It includes both the native Silver fir (*Abies alba*) and the exotic Douglas fir (*Pseudotsuga menziesii*). Both species demonstrate notable resilience to drought, adaptability and production potential in the Central-European conditions [34, 35]. As for Scots pine, despite some reported challenges linked to climate warming and nutrition [36], this species is expected to retain its current share as it often dominates on sandy soils and locations that would not be suitable for other tree species [37].

Broadleaved tree species become increasingly important for the Central-European forestry, as they may benefit from changing climate using sensible management practices [38, 39]. The increased share of broadleaved tree species (Fig. 5), together with the promoted fir species group, translates to over 45 and 50% of the total harvest at the end of simulation period for the Green and the Red scenario, respectively. This is a major change from the recent harvest structure, where coniferous trees represented 89% under “normal” conditions (period 2001–2015) and up to over 95% under current (2018–2021) calamity situation [9].

Finally on tree species, it should be stressed that Norway spruce is far from being doomed for the conditions of Central-European forestry. However, it requires a sensitive selection of suitable locations (higher elevations, waterlogged and humid sites elsewhere) and use of modern silvicultural approaches based on small scale diversified management based on continuous cover forestry systems [40] avoiding clearcuts, using irregular shelterwood and individual tree selection [39, 41]. This would permit individual or group-wise admixture of spruce in structurally and species rich diverse forest ecosystems. This would allow retaining spruce in a reasonable share across a wide elevation gradient (Fig. 7) due to heterogeneity of site conditions also in decades to come, reaching a share within that indicated by the Green and Red scenarios by 2070 (Fig. 5).

The adaptive management as noted above is also required to address the challenges associated with the current extensive clearcuts and their legacy in the expected age structure (Fig. 8). It certainly also includes use of pioneer tree species (SLB, Table 5) aiding effective and diversified recovery of current clearcut areas [42].

Apart from adequate selection of tree species and use of appropriate management, there are other adaptation measures that are proposed with respect to enhancing biodiversity and mitigation potential. One of them is retaining old stands representing set-aside forest area [43], which was tentatively evaluated within the Green scenario. It resulted in a somewhat smaller mitigation effect, quantitatively by about 200 kt C/annually in the last two decades of the simulation period. This is attributed to changes in age structure and hence to NAI due to aging forest stands. This effect was also demonstrated in Fig. 9 showing the example of NAI for spruce and fir, which have contrasting trends of representation by the forest area. Evidently, set-aside areas balance climate and biodiversity considerations, which should be weighed up [44]. Note also that a complete evaluation should include substitution benefits from HWP utilization and substitution function of forestry [44, 45], which was not considered here.

### Additional considerations to the assessed mitigation potential

There is a full range of possible forest-based mitigation activities that may be categorized into protection (avoiding deforestation and degradation), management (forest conservation—set aside areas, harvest regime and other active management), restoration (afforestation/reforestation) and wood use [43]. Our scenarios included protection and management activities, but did not consider restoration and wood use, which is a limitation in this study.

As for afforestation, it brings up clear mitigation benefits, but its impact in Central-European conditions is limited due to the constraints in actual land use. On the contrary, the contribution of HWP for its substitutional (energy and material) mitigation effect [43, 46] may be more important for the countries with yet unrealized potential of wood use and limited domestic wood processing industry. This is because according to the adopted policies, only domestic production and use of HWP can be accounted for the emission targets of European Member States [4, 6]. This applies specifically for the Czech Republic, where the accounted contribution of HWP is relatively important due to the currently increased wood production [22], but may decrease in the near future unless the country increases its domestic production of sawn wood and wood-based panels, and minimizes its substantial export of unprocessed wood. The specific evaluation of HWP contribution along with the current management scenarios is beyond the scope of this study. In the recent decade, the HWP contribution represented in average −1.17 Mt CO_2_ eq., which is a relatively high offset quantity due to a strongly increasing harvest [10]. With a declining harvest for the years to come, HWP contribution is also expected to decline, unless compensated by increased domestic use and decreasing export of roundwood, which is favored in the adopted accounting scheme [4, 5, 24].

Overall, our results suggest that the long-term mitigation potential of Czech forestry can significantly aid offsetting the emissions generated in Agriculture (GHG emission sector including livestock and manure management) once the HPW contribution is included, and forest production remains limited to about 16 Mm^3^ annually. However, it would need to be aided by sensible land use management by other land-use categories within the LULUCF sector [47] that may contribute to meeting this emission target—i.e., at least emission neutral Land sector combining Agriculture and LULUCF, which is currently under consideration in EU for its Member States for the post-2030 period. On the other hand, the retained LULUCF accounting setting using FRL in the revised LULUCF regulation [4, 6] remains problematic and grossly unfair. Also, the LULUCF regulation and its targets remain in apparent conflict with the long-term goals of (at least Central-European) forestry, which certainly cannot be driven by the short-term mitigation targets. On the contrary, it must prioritize adaptation strategies, where climate mitigation by CO_2_ sequestration represents one of the vital, but still residual forest functions. This is increasingly accentuated in the climate smart forest management strategies [48] in contrast to the earlier overly carbon-centric policies such as the former Kyoto Protocol.

It should be noted that our model assessment by CBM did not explicitly consider the possible effect of climate change in the projections. This is because the carbon budget at the country scale is mainly affected by forest management interventions and generally by disturbance regime. Specifically in the country, harvest intensity varied between 15 and 36 mil m^3^ of wood volume annually during the last decade, while on that scale the country-level increment changes were assumed to be an order smaller. This assumption is based on the empirical evidence on tree growth changes in Europe in recent decades (e.g., [49–51]). However, assessing future trends of these drivers and their effects on tree physiology under the likely RCP scenarios (4.5 and 8.5) remain uncertain with respect to complex feedbacks affecting future stand growth and forest productivity in general, and beyond the scope of the current study. Therefore, any possible climate effect on tree increment and tree physiology was neglected (assumed quantitatively neutral) and considered too uncertain to be assessed with respect to complex feedbacks affecting future stand growth in general. Climate effect was considered indirectly by adaptive management selecting appropriate tree species for the local conditions and designing management scenarios with accentuated outlooks for conventional spruce-based forestry and its significant change in coming decades.

## Conclusions

The analysis using CBM projections provides quantitative frame of both the near- and long-term trends in development of forest resources as affected by alternative adaptive management and disturbance scenarios. It is obvious that the near-term emission reduction targets set for Czech forestry under EU LULUCF regulation until 2025 will be difficult to meet (but less so for 2030) considering the extent of the current historically extreme decline of over-represented sensitive spruce-dominated stands. However, the scenarios indicate a possibility of a sustained CO_2_ sink while preserving sizable harvest potential and dramatically changed forest structure in favor of more resilient tree species that would enhance biodiversity and secure provisioning of other expected forest functions to society.

## Methods

### Aim, design and settings of the study

The main aim of the study is to quantify development of forest resources in the Czech Republic for the set of four scenarios of spruce forest stand decline, all including adaptive forest management. The quantification concerns carbon stock with a focus on changes in individual ecosystem carbon pools according to the adopted IPCC methodologies [23, 24] as used for the national GHG emission inventories under UNFCCC. Additionally, key indicators of forest resources are analyzed—specifically the changes in tree species composition and volume/age structure. The spatial domain of the study is the cadastral forest land of the Czech Republic (2 604 kha as of 2018), with a spatial resolution of the NUTS3 regional units (n = 14, Table 3; Fig. 11).

### Modeling tool CBM-CFS3

**Fig. 10:**
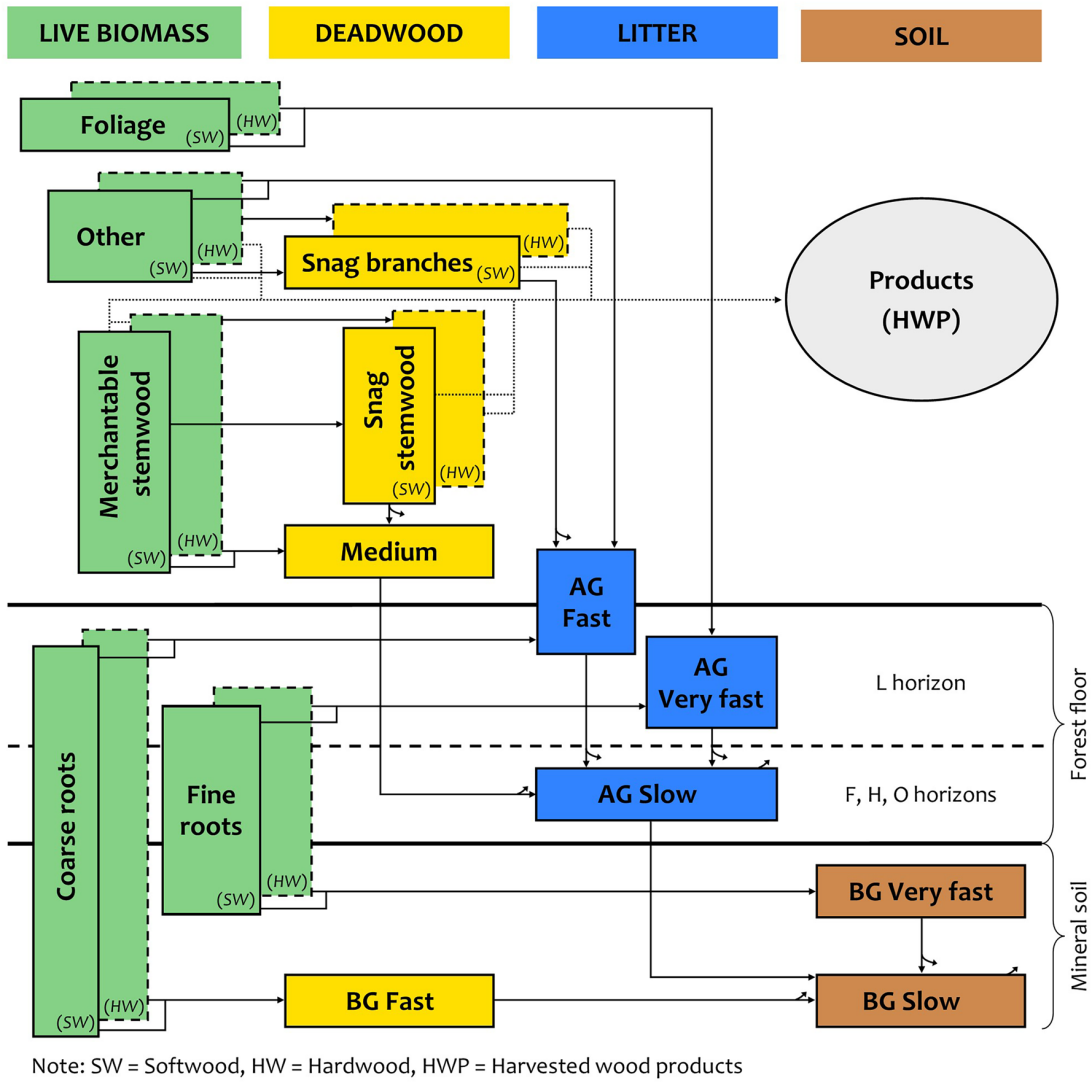
Conceptual diagram of CBM carbon pools and their relationships (straight arrows showing transfers between pools, curved arrows showing transfer to atmosphere), with categorization of relative decay rates (very fast, fast, medium, slow), for softwoods and hardwoods, which are distinguished by double-frames for five live biomass pools and two deadwood pools (adapted from Kull et al. 2019). This makes the total number of specific carbon pools in the model equal to 21. The transfers to wood products (HWP) are also shown

This study uses a specifically calibrated modeling tool called Carbon Budget Model of the Canadian Forest Sector (CBM-CFS3 v. 1.2, here also denoted as CBM [13, 14];). CBM represents a flexible modelling framework that has also been applied for forest ecosystem analyses and carbon-accounting purposes in other European countries [15, 16]. Pilli et al. [20]s applicable to European conditions, which was also used as a basis for this country-specific application. CBM is an inventory based, yield-data driven model that simulates the stand- and landscape-level carbon (C) dynamics of above- and below-ground biomass, and dead organic matter (DOM) including soil [14]. In its spatial representation beyond single stands, it can be flexibly set up to represent administrative and climate regions. CBM uses in total 21 carbon pools, which are linked to IPCC carbon pools as shown in Table 1 and in the conceptual diagram in Fig. 10.

**Table 1:** IPCC carbon pools and their equivalents in CBM (adapted from [14])

IPCC carbon pool	Pool name in CBM-CFS3	Description
Living Biomass		
Aboveground biomass	Merchantable stemwood and bark	Live stemwood of merchantable size* plus bark
	Other wood and bark	Live branches, stumps and small trees including bark
	Foliage	Live foliage
Belowground biomass	Coarse roots	Live roots, 5 mm and larger diameter
	Fine roots	Live roots, less than 5 mm diameter
Dead organic matter		
Deadwood	Snag stems DOM	Dead standing stemwood of merchantable size incl. bark
	Snag branches DOM	Dead branches, stumps and small trees
	Medium DOM	Coarse woody debris on the ground
	Belowground fast DOM	Dead coarse roots (diam. 5 mm and more) in mineral soil
Litter	Aboveground fast DOM	Fine and small woody debris and dead coarse (submerch. size) roots in the forest floor
	Aboveground fast DOM	F, H and O horizons
	Aboveground very fast DOM	L horizon incl. foliar litter and dead fine roots (< 5 mm diam.)
Soil		
Soil organic matter	Belowground very fast DOM	Dead fine roots (< 5 mm diam.) in the mineral soil
	Belowground slow DOM	Humified organic matter in the mineral soil

^*^Merchantable size wood limit uses the Czech standard of min. 7 cm in diameter

To use CBM-CFS3 in the Czech national circumstances, the European Archive Database as prepared by the Joint Research Centre [20] was modified to include the locally applicable biomass allometry functions for beech, pine, spruce, oak and birch [52–57]. The calibration process was based on the sample-based landscape inventory data [58] and CBM procedure as described by [59]. The fitting procedure and applicable species-specific parameters are described in [21].

**Fig. 11:**
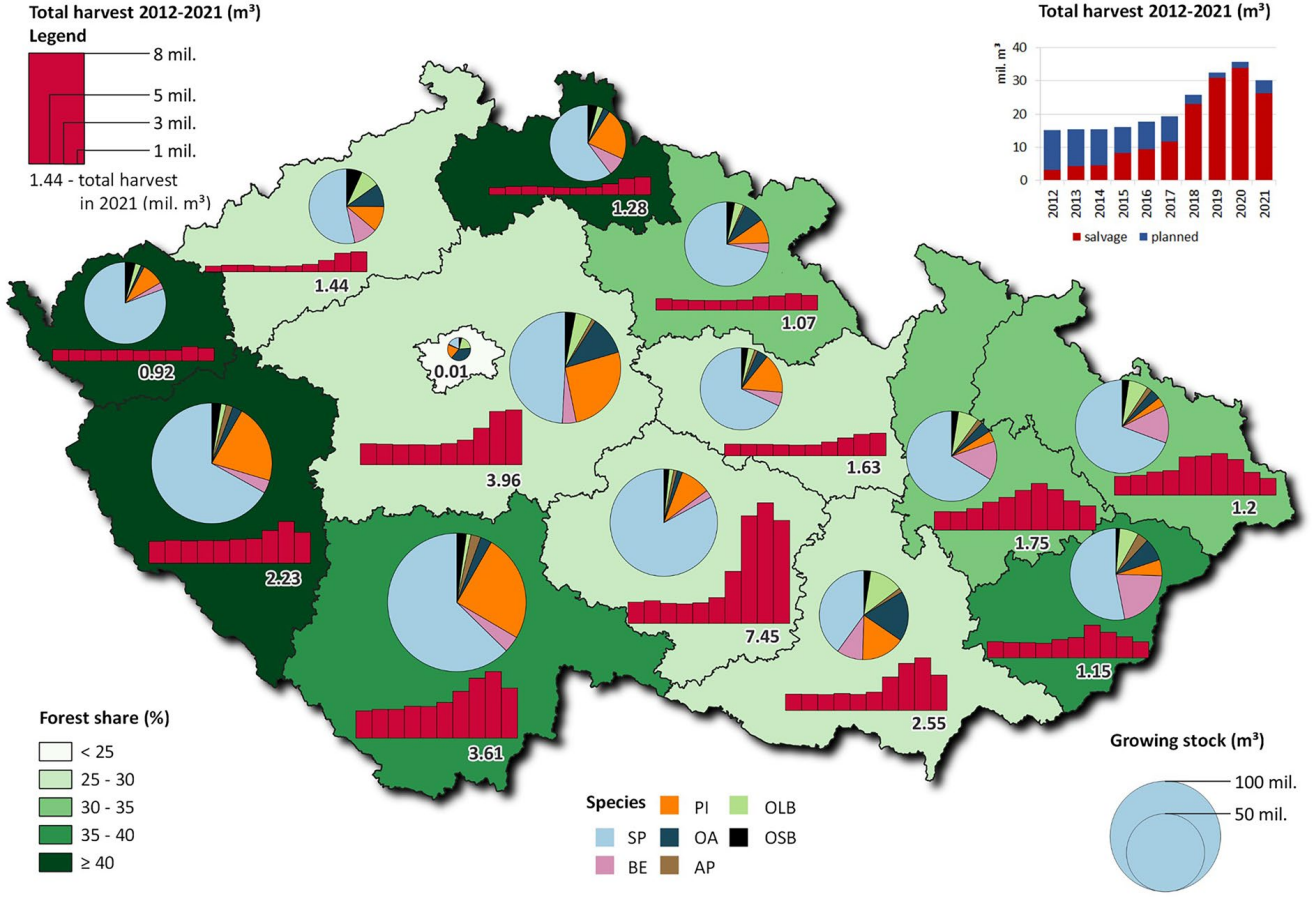
Simulated domain—forest area (share as of 2021) the Czech Republic at NUTS3 regional resolution. Green color grade shows forest area share by region, pie graphs show share of species groups (forest types) by volume with a size corresponding to growing stock volume, red bars give harvest level for period 2012–2021 to demonstrate progress and magnitude of the recent drought-induced bark-beetle calamity. The graph up-right gives the reported total harvest distinguishing planned and sanitary interventions at the country level

The model was initialized using the national data on the Czech forest resources as of 2018 (Table 5) using the centralized database of Forest Management Plans (FMP) administered centrally by Forest Management Institute (FMI), Brandýs n. Labem. These data included information on growing stock volume by age classes and tree species categorized in seven groups representing forest types (Table 5) and 14 regional (NUTS3) spatial units (Table 3, Fig. 11). Net (current) annual increment (NAI) applicable to forest types and their productivity as of 2018 was also provided by FMI based on the national Growth and yield tables [60]. CBM uses merchantable volume data over age to simulate growth. The entire CBM growth concept is described in detail by Kurz et al. [14] and Pilli et al. [15], and its national application described in [21]. Turnover rates and transfer to DOM carbon pools are based on the values published for CBM in the European CBM-specific database [20], with stem biomass mortality derived from the Czech NFI [61]. The information on biomass turnover, designated DOM pools and litter transfer rates as applied in CBM is provided in [21].

The past and future carbon dynamics of forest ecosystems are controlled in the model by a set of prescribed management interventions and natural disturbances. Scheduling the timing of timber harvest (thinning, salvaging, final cut) for each species group is organized in the model input file and disturbance event tables, which define the minimum forest age and biomass for clearcut, minimum and maximum age for thinning and the thinning interval. The set of disturbances (management interventions) used here are described in Table 2, the corresponding transfer matrices are used similarly as published in [21]. Specifically for this study, extraction of harvest residues was set to 20 and 25% following sanitary (Dist. 3a in Table 2) and planned (Dist. 4 in Table 2) harvest, respectively. It should be understood that forestry uses planned interventions such as thinning and final cut (Dist. 2 and Dist. 4 in Table 2), as well as sanitary harvest interventions (Dist. 3a, Dist. 3b in Table 2). According to the forestry legislation of most Central-European countries, the latter must be prioritized on the account of the planned activities in case of calamities to minimize their environmental impact and limit spreading. Hence, the ratio of sanitary and planned harvest (Fig. 11) is a solid indicator of stability of forests and forest management.

**Table 2:** Description of the individual disturbance types that are entered in the input file, all in mass unit of carbon (t C) except Dist. 6 and Dist. 8 that represent interventions defined by area (ha)

Disturbance type ID	Disturbance name	Description
Dist. 2	Thinning	Commercial thinning of merchantable trees in age classes 2–5 (species-dependent) resulting in a 10–30% reduction in biomass carbon
Dist. 3a	Salvage with clear-cut	Salvage clear-cut logging of 100% of merchantable trees on areas with both abiotic and biotic disturbance. A fraction of harvest residues may be burned
Dist. 3b	Salvage without clear-cut	Selective salvage logging of merchantable trees in small patches with abiotic and/or biotic disturbance that does not result in open clear-cuts
Dist. 4	Clear-cut harvesting without salvage	Final commercial felling of merchantable trees, 5% of trees left aside as seed trees enhancing biodiversity. A small portion of harvest residues may be burned
Dist. 5	Clear-cut with slash-burn	Logging of 85% of merchantable trees followed by the burning of slash. This disturbance is only used for the initialization of dead organic matter pools
Dist. 6	100% mortality	Death of standing trees due to drought and/or bark-beetle attack, representing transfer of living biomass to snag stemwood (in area units)
Dist. 7	90% mortality	Harvest of snag stemwood after previous mortality of standing trees due to drought and/or bark-beetle attack
Dist. 8	Reforestation	Reforestation of forest stands after the previous mortality of tree layer (in area units) - not involving any land-use conversion

### Simulated domain

The simulated domain is the forest area in the Czech Republic with a NUTS3 regional resolution (Fig. 11). The total area of the country is 7 887 kha, the simulated domain equals totally 2 604 kha of forest land representing timberland (including the clearcut areas) according to the Czech national cadastral system as of 2018. The fraction of unstocked cadastral forest land (63.6 kha in 2018) is not included. The share of cadastral forest land at the country-level was 34%. All elaborated scenarios assume a constant forest area for the entire projection period 2018–2070, any afforestation and deforestation events representing a possible land use change are not considered. The list of the 14 NUTS3 regions and the key region-specific information is included in Table 3.

**Table 3:** List of NUTS3 regions with basic information and scenario assumptions

NUTS3 name	Code	NUTS 3 area [kha]	Forest cover* [%]	Enhanced species	Average altitude [m A.S.L.]	Pessimistic scenario assumptions	
						End of disturbance episode** [year]	Calamity spread***
Hl. m. Praha	CZ010	50	9.6	Oak	286	2037	No
Středočeský	CZ020	1093	26.9	Oak	414	2025	Yes
Jihočeský	CZ031	1006	37.0	Fir	631	2030	Yes
Plzeňský	CZ032	765	39.8	Fir	609	2035	Yes
Karlovarský	CZ041	331	42.6	Fir	684	2023	Yes
Ústecký	CZ042	534	29.7	Oak	516	2030	Yes
Liberecký	CZ051	316	43.2	Oak	522	2036	Yes
Královéhradecký	CZ052	476	30.6	Oak	548	2047	Yes
Pardubický	CZ053	452	29.0	Oak	486	2033	Yes
Vysočina	CZ063	680	30.0	Fir	574	2022	No
Jihomoravský	CZ064	719	26.8	Oak	378	2021	No
Olomoucký	CZ071	527	34.4	Fir	592	2032	No
Zlínský	CZ072	396	39.2	Oak	503	2031	No
Moravskoslezský	CZ080	543	34.7	Fir	591	2036	No

^*^Forest cover share

^**^The region-specific end of disturbance episode is set for the pessimistic Red, Black, and Black with repetition scenarios

^***^The spread of bark-beetle calamity since the last observation (2021) is considered for the Black and Black with repetition scenario

### Management scenarios

Four scenarios of spruce forest dieback and adaptive forest management (Green, Red, Black, Black rep.) were developed to analyze potential alternatives for the ongoing bark-beetle calamity and the effects of implemented sanitary measures (Table 4). All scenarios adhere to the current national forest adaptation policy, which is applied with specific intensity to reduce and restructure vulnerable spruce-dominated stands. However, they differ in their projections regarding the end of the current disturbance episode, in harvesting regime for the subsequent decades until 2070, and, indirectly, in intensity of tree species change.

**Table 4:** Summary of the tested forest management scenarios

Scenarios	Description	Target removal [Mm^3^/yr]*	Spread of the recent calamity**	Calamity reoccurence ***	Preserving old trees ****
Green	Optimistic scenario with the rapid attenuation of the recent calamity Species change promoting fir and broadleaves	17	No	No	Yes
Red	Pessimistic scenario with a progressive decrease of the recent calamity until 2030. Species change promoting fir and broadleaves	16	No	No	No
Black	Pessimistic scenario with a slight spread of the recent calamity until 2030 followed by its gradual decline. Species change promoting fir and broadleaves	16	Yes	No	No
Black rep	Pessimistic scenario based on the Black, with a 2-year calamity recurrence every 10 years. Species change promoting fir and broadleaves	16	Yes	Yes	No

^*^Removal target in Mm^3^ merchantable wood volume under bark

^**^Region-specific spread of bark-beetle calamity with a 20% increase in sanitary logging compared to 2021 level

^***^Reoccurring calamity as of 2018/2019 every decade

^****^Additional biodiversity measure

The Green scenario represents an optimistic development of the bark-beetle calamity, with a rapid decline and a stable annual harvest volume of approximately 17 Mm^3^ of merchantable wood under bark after the current disturbance episode ends. Conversely, the other scenarios (Red, Black, and Black rep.) take a more pessimistic approach, assuming a gradual and slower decline in the ongoing bark-beetle calamity, with an annual harvest volume of around 16 Mm^3^ once the current disturbance subsides.

The harvesting regimes in each scenario differ primarily in terms of salvage logging, which is based on the observed sanitary harvest [9] in the NUTS3 regions during the recent years from 2012 to 2021 (Fig. 11). The proportion of salvage logging has increased over time as a response to the advancing bark-beetle calamity and the decline of spruce forest stands. Once the calamity subsides, the harvest demand aligns with the sustainable logging potential, which is determined by the standing stock volume across different age classes and forest types (groups of tree species). During the period of ordinary planned management, the amount of sanitary logging does not exceed one-third of the total harvest. This proportion is consistent with the values observed prior to the current outbreak of the calamity. The aim is to maintain a balance between regular harvesting activities and the need for sanitary logging to address the impacts of the bark-beetle calamity. All scenarios include identical assumptions of a moderate extraction intensity of harvest residues. It was set to 20 and 25% for salvage (Dist. 3a in Table 2) and planned (Dist. 4 in Table 2) harvest, respectively.

The Green scenario is based on the observation that, as of 2021, that the calamity reached its maximum in most NUTS3 regions in 2020 or earlier, and a gradual decline in harvest intensities is observed in subsequent years. The histograms of harvest volumes at the NUTS3 regional resolution in Fig. 11 indicate that the current disturbance episode, respectively the harvest intensities, follow a normal distribution. Using the non-linear regression model, the parameters of normal distribution of the total harvest volumes for each NUTS3 region were estimated on the yearly data observed from 2012 to 2021 (Fig. 11). Subsequently, the total harvest volumes in regional resolution for the period of 2022–2030 were extrapolated based on the fitted curves. Next, the tree species and logging type composition of harvest demand in this part of projection period were determined by using exponential interpolation of the corresponding harvest shares between 2021 and 2031. From 2030 onwards, the harvest demand was determined according to the logging potential and sustainable harvest for individual tree species groups and NUTS3 regions at annual time step. Under this scenario, the guiding merchantable total annual harvest volume under this scenario is about 17 Mm^3^. Furthermore, as one of the widely advocated adaptive measures [52], old-growth forest stands, namely 120- and 140-year-old and older coniferous and broadleaved stands, respectively, will be retained without sanitary logging and final cut interventions after 2025.

The Red scenario assumes a continued decline of the vulnerable spruce stands following the current calamity, with a specific setting for the end of the disturbance episode according to the available spruce growing stock in each given NUTS3 region. During this part of projection period, the harvest demand is set according to the observed harvest intensities for a given disturbance type (Dist. 2, Dist. 3a, Dist. 3b and Dist. 4; Table 2), tree species group and NUTS3 region in 2021. Once the harvest limit of mature spruce stands is reached in each NUTS3 region, a transition to a sustainable harvest regime according to the logging potential for individual tree species groups is assumed. The assessed termination of the current bark-beetle calamity and the decline of spruce forest stands by the NUTS3 regions are provided in Table 3. Under this scenario, the guiding merchantable total annual harvest volume is about 16 Mm^3^.

The Black scenario envisions the further spread of the current calamity in the NUTS3 regions where sanitary logging has not yet reached its peak. For these regions, sanitary logging of spruce is increased by 20% (compared to 2021 levels) since 2022, continuing until the harvest limit of mature spruce stands is reached. Subsequently, the transition to a regime according to the logging potential is expected. For all the remaining NUTS3 regions, the harvest demand for the sustained calamity period is set as in the Red scenario, i.e., according to harvest volumes observed in 2021. The length of the current disturbance episode with the spread of calamity is similar to the time settings in the NUTS3 regions as in the Red scenario. Once the limit of mature spruce stands in each NUTS3 region is reached, a subsequent transition to a sustainable harvest regime according to the logging potential for individual tree species groups is assumed. Under this scenario, the guiding merchantable total annual harvest volume is about 16 Mm^3^.

Similar to Black scenario, the Black scenario with repetition (Black rep.) envisions the further spread of the current calamity in NUTS3 regions where the calamity has not yet culminated as of 2021. The harvest demand intensities over the length of the projection period, when the spread of bark-beetle calamity occurs, are set to be like in the Black scenario. Furthermore, 2-year disturbance episodes with bark-beetle infestation of spruce stands occur regularly every 10 years from the end of the 2030s until the end of the projection period. The annual logging intensities of spruce stands during a 2-year episode are set as average harvest volumes from 2018 and 2019. During the period between 2-year calamities, the harvest of spruce stands takes place according to the logging potential. For other forest types, a transition to a regime according to the logging potential is assumed once the current calamity subsides. Compared to the Red and Black scenario, the planned harvest demand (Dist. 2 and Dist. 4) for other tree species (except fir) is increased by 10%, 15% and 20%, in the period of 2038–2047, 2048–2057 and 2058–2070, respectively.

All four scenarios include a species change as a key adaptation measure. The species change occurs when wood is harvested either by sanitary logging interventions (Dist. 3a, 3b; Table 2) or following the planned final cut (Dist. 4; Table 2). The species change associated with sanitary logging assumes a replacement of spruce with beech (20%), fir and oak (10% and/or 30% depending on elevation of the NUTS3 region as in Table 2), long-lived broadleaves (LLB, Table 5, 20%) and short-lived broadleaves (SLB, Table 5, 20%). The species change associated with planned final cut assumes that 50% of the spruce species area is replaced by fir (10%), beech (20%) and OLB (20%) species groups.

**Table 5:** Forest types used to categorize tree species and category of unprocessed dead standing spruce trees and clearcut areas

Forest type	Acronym	Main species	Area [kha]	Area share (%)	Volume share (%)
Spruce	SP	*Picea abies* (L.) Karst	1 293	49.6	59.5
Pine	PI	*Pinus sylvestris* L., *Pinus nigra* Arnold	528	20.2	19.9
Beech	BE	*Fagus sylvatica* L	225	8.6	6.7
Oak	OA	*Quercus petrae* (Matt.) Liebl., *Q. robur* L	194	7.4	5.4
Long-lived broadleaves	LLB	*Tilia cordata* Mill., *Tilia platyphyllos* Scop., *Fraxinus excelsior* L., *Acer pseudoplatanus* L., *Carpinus betulus* L	159	6.1	4.0
Short-lived broadleaves	SLB	*Betula pendula* Roth., *Alnus glutinosa* (L.) Gaertn., *Populus spp.*, *Alnus incana* (L.) Moench	139	5.3	2.6
Fir	AA	*Abies alba* Mill., *Pseudotsuga menziesii* (Mirb.) Franco	37	1.4	1.5
Clearcut area	–	Temporarily unforested area, e.g., after clear-cut	31	1.2	–
Spruce snag	SPx	Unprocessed standing dead spruce forest stand (dead due to drought stress and bark beetle)	5	0.2	0.3
Total			2 610	100	100

Excluded is the unstocked cadastral forest area (forest roads, nurseries etc.) representing a share additional 2.4% (63.6 kha). This makes the total cadastral forest area 2 673 kha in the country, of which timberland makes up 2 610 kha in 2018

The specific forest type representing standing dead spruce stands (SPx, Table 5) that remain unprocessed after bark beetle infestation due to insufficient logging capacity, was treated exclusively for the recent period of 2018–2021 as follows. Aboveground biomass of dead merchantable stems transits to stem snag pool, while carbon in other biomass pools is converted to the respective DOM pools. The postponed harvest of unprocessed dead stands is carried out in the following year after the dieback. Specifically, 90% of the previous year's unprocessed merchantable volume is removed from the snag stemwood pool in the following year. The rest of unprocessed volume is left to decomposition. The unprocessed volumes were determined in the NUTS3 resolution based on the available national statistic [9] on unprocessed dead trees for 2018–2021.

## Data Availability

The datasets used and/or analyzed during the current study are available from the corresponding author on a reasonable request.

## Abbreviations

CBM: Abbreviation from the CBM-CFS3
CBM-CFS3: Carbon Budget Model of the Canadian Forest Sector
CO_2_: Carbon dioxide
DOM: Dead organic matter
FMI: Forest Management Institute
FMP: Forest management plans
FRL: Forest reference level (under EU LULUCF regulation)
FMRL: Forest management reference level (under Kyoto Protocol)
GHG: Greenhouse gasses
HWP: Harvested wood products
IPCC: Intergovernmental panel on climate change
LULUCF: Land use, land-use change and forestry
NAI: Net annual increment
NFI: National forest inventory
NIR: National inventory report for reporting emissions under UNFCCC
NUTS3: Nomenclature of Territorial Units for Statistic, level 3
RCP: Relative concentration pathway
UNFCCC: United Nations Framework Convention on Climate Change
